# Sex-specific differences in preclinical models of advanced chronic liver disease and portal hypertension

**DOI:** 10.1186/s13293-025-00721-8

**Published:** 2025-06-03

**Authors:** Peio Aristu-Zabalza, María Andrés-Rozas, Zoe Boyer-Díaz, David P. Al-Adra, Douglas Maya-Miles, Sergi Guixé-Muntet, Anabel Fernández-Iglesias, Jordi Gracia-Sancho

**Affiliations:** 1 Barcelona Liver Bioservices SL, Barcelona, Spain; 2 Liver Vascular Biology Lab, IDIBAPS Biomedical Research Institute-Hospital Clínic de Barcelona, Rosselló 149, 08036 Barcelona, Spain; 3 Facultat de Medicina i Ciències de la Salut, Universitat de Barcelona (UB), Barcelona, Spain; 4 University of Wisconsin School of Medicine and Public Health, Madison, WI USA; 5 CIBEREHD, Madrid, Spain; 6 SeLiver Group, IBIS & Virgen del Rocío Hospital, Seville, Spain

**Keywords:** Hepatic hemodynamic, Sinusoidal cells, Gender, Liver transcriptomics, Cirrhosis

## Abstract

**Background:**

Chronic liver disease is a major health concern, but sex-specific differences in its pathophysiology remain unclear. Preclinical studies have predominantly used male animals, limiting findings' relevance to both sexes. This project aimed to explore sex differences in cirrhosis and portal hypertension (PH) in rats, and to find similarities in human samples for translational relevance.

**Methods:**

Advanced chronic liver disease (ACLD) was induced in male and female Sprague–Dawley rats using thioacetamide (TAA, 250 mg/kg; 12 weeks) or bile duct ligation (BDL; 28 days). Healthy rats served as controls (n = 11–18/group). We assessed in vivo hepatic and systemic hemodynamic parameters, hepatic microvascular function, and hepatic transcriptomic analyses, including sex-specific differences in cellular composition using gene deconvolution (n = 5/group). Two human sample cohorts were compared to preclinical data for translational insights.

**Results:**

Both animal models showed PH. TAA males had similar portal pressure (PP) to females (14.2 vs 14.1 mmHg), but BDL males had significantly higher PP than females (14.5 vs 12.5 mmHg; p = 0.003). No differences were observed in hepatic microvascular function. In the BDL model, females had more fenestrae and porosity, and less fibrosis. Transcriptomic analysis revealed that TAA males had dysregulated metabolic pathways, while females had deregulated genes in hormone signaling. In the BDL model, males showed higher deregulation in platelet activation, protein degradation, vesicular transport, and disease-related pathways. Gene deconvolution showed males had a more specialized endothelial phenotype basally, with more changes in endothelial and macrophage phenotypes after injury. In MASLD patients, men had dysregulated metabolic pathways, while women showed deregulation in fibrosis, extracellular matrix, and endocrine regulation. In HBV patients, men had more dysregulation in fibrosis, inflammation, and immune response. Female MASLD patients had more activated hepatic stellate cells, and greater loss of endothelial phenotype compared to men.

**Conclusions:**

This study highlights sex-dependent molecular differences in the pathophysiology of cirrhosis in two preclinical models. Further research in preclinical and human liver disease is essential to develop safe and effective treatments for ACLD in both sexes.

**Supplementary Information:**

The online version contains supplementary material available at 10.1186/s13293-025-00721-8.

## Highlights

- Animal models showed portal hypertension. BDL males had significantly higher portal pressure and fibrosis than females, while BDL females showed increased sinusoidal fenestration and reduced disease severity.
- Transcriptomic analysis in TAA models revealed dysregulated metabolic pathways in males, while females displayed alterations in hormone signalling. In BDL models, males showed higher deregulation in platelet activation, protein degradation, vesicular transport, and disease-related pathways.
- In MASLD patients, transcriptomic data showed males with dysregulated metabolic and inflammatory pathways, while females exhibited alterations in fibrosis, extracellular matrix remodelling, and endocrine regulation, corroborating preclinical findings.
- Gene deconvolution in animal models revealed sex-specific changes in hepatic endothelial and macrophage phenotypes, with males undergoing greater shifts after liver injury.
- In MASLD patients, gene deconvolution showed females with greater endothelial phenotype loss and hepatic stellate cell activation than males, aligning with fibrosis-related findings in transcriptomic data.

**Supplementary Information:**

The online version contains supplementary material available at 10.1186/s13293-025-00721-8.

## Plain language summary

Chronic liver disease is a serious global health issue, but men and women experience it differently. Despite this, most preclinical research has focused on male animals, creating a gap in understanding how the disease develops and progresses in females. Our study aimed to address these sex-based differences using two preclinical models of advanced liver disease in male and female rats, and to compare these findings with patients. We identified significant differences between male and female rats with liver disease. In one model, male rats exhibited higher portal pressure and more extensive liver fibrosis, while females displayed milder disease and healthier liver structures. Analysis of genetic activity in liver tissue revealed sex-specific patterns: males showed alterations in metabolic pathways, while females exhibited changes related to hormone signalling. These findings were consistent with observations in human liver samples, where men demonstrated greater inflammation and metabolic dysfunction, and women showed more pronounced fibrosis and vascular remodelling. Furthermore, we analysed specific liver cell types and found that male rats experienced greater changes in certain cells after injury, potentially contributing to worse outcomes. These insights underscore the importance of including both sexes in research to develop safer and more effective treatments for advanced liver disease.

**Supplementary Information:**

The online version contains supplementary material available at 10.1186/s13293-025-00721-8.

## Background

Cirrhosis represents the final stage of chronic liver diseases (CLD), consequence of a prolonged combination of scarring (fibrosis), vascular dysfunction and occlusion, parenchymal extinction, and nodular regeneration. Main underlying aetiologies leading to CLD include alcohol use disorders, steatohepatitis, viral infections, and autoimmune and genetic diseases [1]. Portal hypertension (PH) is a severe syndrome associated with advanced CLD, characterized by an increase in hepatic venous pressure gradient (HVPG) which is clinically significant when exceeding 10 mmHg, accompanied by significant morbidity and mortality. The primary factor in the development of PH is a marked increase in the intrahepatic vascular resistance (IHVR) to portal blood flow, occurring early during CLD due to profound phenotypic alterations in sinusoidal cells. These alterations include hepatocyte necroptosis, hepatic stellate cells (HSC) activation, liver sinusoidal endothelial cells (LSEC) capillarization, and macrophage recruitment, altogether leading to fibrosis, vascular remodelling, and increased hepatic vascular tone [2, 3]. Despite extensive preclinical and clinical research efforts developed in recent decades, an effective clinical treatment for elevated IHVR in PH is still lacking.

Animal models are essential for enhancing our understanding of human pathogenesis, allowing researchers to identify therapeutic targets and evaluate new drugs [4]. However, in most diseases, preclinical research seems to be losing sight of its fundamental objective. This omission primarily stems from its failure to encompass the diversity of patient populations, leaving women underrepresented in the process. The inclusion of both sexes in animal studies is crucial, as it can yield contrasting results that highlight the importance of sex differences in disease mechanisms and treatment responses [5]. For instance, including both sexes in animal studies have revealed different or even contrasting effects, with some drugs enhancing memory in females while reducing it in males, or reducing fat accumulation in females but increasing it in males [6, 7]. Nevertheless, many preclinical studies continue to rely heavily on male animal models, introducing a noticeable bias toward male perspectives, thus clinical trials often reveal a higher incidence of adverse effects in women compared to men [8]. In the field of hepatology, research has shown that oestrogen protects female mice from liver injury caused by omega-3 PUFAs in the context of acetaminophen (APAP) toxicity, while males lack this protection. Additionally, women with NAFLD respond more favourably to pioglitazone compared to men, showing a greater reduction in liver fat content, which underscores the importance of considering sex differences in treatment efficacy [9, 10].

These observations underscore the necessity of a more inclusive approach in preclinical research, recognizing the importance of sex and highlighting differences in prevalence, pathophysiology, and clinical presentations. A holistic understanding of these distinctions is vital for developing effective and equitable medical interventions [11]. Therefore, this study aimed to comprehensively characterize cirrhotic PH in male and female rats using two widely used experimental models (chronic thioacetamide (TAA) and bile duct ligation (BDL)) along with healthy rats, to understand sex-related complexities and potential implications. Translational relevance was attained by characterizing well-defined cohorts of patients. This study provides the first detailed comparative characterization of liver disease in preclinical models of advanced CLD.

## Methods

### Animal models of advanced chronic liver disease

Advanced chronic liver disease (ACLD) was induced in male and female Sprague–Dawley rats following two well-known protocols. Firstly, rats (150 g, Janvier) underwent repeated administration of thioacetamide (Sigma) injected twice a week for a total of 12 weeks. TAA was previously dissolved in a saline solution at a concentration of 125 mg/ml and given intraperitoneally at a dosage of 250 mg/kg body weight, as previously described [4, 12, 13].

Secondly, secondary biliary cirrhosis with intrahepatic PH was generated in male and female Sprague–Dawley rats (250–275 g, Janvier) by performing common bile duct ligation [4, 14]. Briefly, rats were induced to anaesthesia using inhaled isoflurane (Isovet) and the common bile duct was occluded using double ligatures made from 5-0 silk thread. Subsequently, the section of the bile duct located between the two ligatures was excised and, after 28 days, the animals were studied.

Animals with ACLD were used for in vivo haemodynamic studies, ex vivo hepatic microvascular function, molecular determinations, and for RNA-sequencing. Healthy rats of both sexes were also included in this study (Male rats, 400–450 g, and female rats, 260–280 g, Janvier). All animals in our study fell within the adult stage (12–17 weeks), ensuring comparability in terms of physiological development.

The rats were housed in pairs, separated by sex but kept in the same room, following a 12:12-h light–dark cycle, within controlled animal facilities at the Institut d'Investigacions Biomèdiques August Pi i Sunyer (IDIBAPS). All procedures were sanctioned by the Laboratory Animal Care and Use Committee of the University of Barcelona and adhered to the guidelines of the European Community for safeguarding animals used in experimental and other scientific pursuits (EEC Directive 86/609).

### In vivo hemodynamic analysis

Before the study, animals underwent a fasting period lasting 8–12 h to prevent any disruptions in splanchnic hemodynamics. Rats were induced into anaesthesia using inhaled isoflurane (Isovet).

Measurement of mean arterial pressure (MAP) and heart rate (HR) was accomplished by inserting a heparinized p50 catheter (Portex) into the femoral artery for MAP and through the ileocolic vein for portal pressure (PP), both linked to a pressure probe. To ascertain portal blood flow (PBF), a specific nonconstrictive perivascular ultrasonic transit-time flow probe (Transonic Systems Inc.) was employed. In order to prevent interference from portal-collateral blood flow, PBF measurements were taken after the bifurcation of the splanchnic vein, close to the liver entrance. Pressure and flow probes were connected to a Powerlab (4SP), and the data was recorded using Chart v5.5.6 software. Hemodynamic parameters were gathered after a stabilization period lasting 20 min [14]. Once the hemodynamic measurements were completed, blood and tissue samples were collected for subsequent molecular and histological analysis.

### Ex vivo analysis of hepatic microvascular function

In a sub-group of animals, and after in vivo hemodynamic assessment, the liver was isolated, and both the portal and hepatic veins were cannulated with p240 catheters, which were then connected to a controlled perfusion system. In accordance with the procedure outlined in [15], the liver was perfused with Krebs buffer at a consistent flow rate of 35 mL/min and the perfusion portal pressure (PPP) was recorded using a pressure probe connected to a Powerlab (4SP). Following a stabilization period lasting 20 min, the intrahepatic microcirculation was pre-constricted by introducing the α1-adrenergic agonist methoxamine (10^−4^ M; Sigma) into the reservoir. Subsequently, the liver microvascular function was evaluated by studying concentration–response curves in response to incremental doses of acetylcholine (10^–6^, 10^–5^, and 10^−4^ M; Sigma) [14]. After the completion of the study, samples of liver tissue were collected.

### Liver histology

Liver tissue samples were preserved in 4% formaldehyde (Sigma), then processed by embedding in paraffin, cutting into sections, and staining with 0.1% Sirius Red in a picric acid aqueous solution (Sigma). Subsequently, ten images from each slide were analysed using a microscope (OLYMPUS BX51) equipped with a digital camera, and the area stained in red was quantified through ImageJ 1.50e software (NIH). The results are expressed as the mean percentage of fibrosis per total area.

### Biochemical analysis

Serum levels of aspartate aminotransferase (AST, U/L), alanine aminotransferase (ALT, U/L), gamma-glutamyl transpeptidase (GGT, U/L), albumin (g/L), urea (mg/dL), uric acid (mg/dL), creatinine (mg/dL), and total bilirubin (mg/dL) were determined using reflectance spectrophotometry at Laboratorio Echevarne S.A. Serum levels of oestradiol (pg/mL; E2 Rat ELISA kit Fine Test, Cat. No. ER1507), progesterone (ng/mL; Pg Rat ELISA kit Fine Test, Cat. No. ER1255) and luteinizing hormone (mlU/mL; LH, Rat ELISA kit Fine Test, Cat. No. ER1123) were determined using ELISA kit.

### Sinusoidal characterization using scanning electron microscopy

In a sub-group of animals (n = 5 per group), after obtaining in vivo hemodynamic data, the livers were perfused via the portal vein with a solution containing 2.5% glutaraldehyde and 2% paraformaldehyde and fixed overnight at 4 °C. Samples were washed 3 times with 0.1 M cacodylate buffer. Liver sections were fixed with 1% osmium in cacodylate buffer, dehydrated in ethanol, and dried with hexamethyldisilazane. From each animal, six blocks were chosen randomly, mounted onto stubs, and coated with a layer of gold using sputter coating. Using a Jeol 6380 Scanning Electron Microscope (JEOL Ltd), ten images per animal were captured at a resolution of 15,000x. Liver sinusoidal fenestrations were quantified using ImageJ 1.50e Software (NIH) [14].

### RNA sequencing

Liver tissue RNA (n = 5 per group) was isolated using Trizol (Life Technologies), and quality evaluated using RNA-TS4200 (n > 10). Samples with sizes around 300 bp and RIN > 8 were used for RNA sequencing. mRNA strand-specific RNA libraries were generated using 300 ng of total RNA with the Illumina® Stranded mRNA Prep Ligation kit following the manufacturer’s instructions. Libraries were sequenced on an Illumina NextSeq2000 (Illumina, Inc.) in paired-end mode with a read length of 2 × 50 bp. Sequencing was performed on the NextSeq 2000 System with P2 Reagent Hit (100 cycles).

RNA reads were mapped against the Rattus norvegicus genome (Rn6.0) for rat liver tissue. Non-biological variability was normalized using the quantile method and limma-voom transformation. Differential expression between groups was evaluated using moderated t-statistics. All deregulated genes were considered significant when the fold change was > 1.5 and p-value < 0.05. RNA sequencing data are deposited and stored in Gene Expression Omnibus.

Principal component analysis (PCA) of the normalized expression data was performed to explore the overall variance and assess the clustering of the samples according to their respective experimental conditions. PCA was performed using DESeq2 for R, and the first two principal components were plotted to visualize the distribution of samples, identifying patterns and similarities between groups.

Deregulated genes were uploaded to g:PROFILER Bioinformatics Resources, a web server for functional annotation and enrichment analyses of gene lists [16]. Gene set enrichment analyses (GSEA) was used for functional enrichment. Transcriptomic data from patient cohorts (GSE: 84044 and 162694) were processed following the methodology previously outlined for bioinformatic analyses (Supplementary Table 1). From the pathways identified using g:GPROFILER, candidate genes with potential roles in ACLD were selected and analyzed using the GTEx portal, a database that compares gene expression across various human tissues [17].

### Gene deconvolution

Relative fractions of the different hepatic cell subpopulations were estimated by gene deconvolution. Briefly, single-cell sequencing data from the literature [18, 19] were reanalyzed as described in the original papers. The unlabelled sinusoidal phenotypes in the rat dataset were named as the corresponding clusters from the human dataset, based on similarities in gene expression. At this point, the clusters described in the single-cell sequencing data were labelled according to their main cell type and subclassification (e.g. endothelial cells, including LSEC and scar-associated LSEC; mesenchymal cells, including quiescent HSCs and myofibroblast-like cells; or macrophages, including Kupffer cells (KCs) or scar-associated macrophages). The labelled data was then uploaded to CIBERSORTx [20] in order to generate signature matrices. These matrices detail the most characteristic gene signatures for each of the cell subtypes. Using these matrices as input, we then performed gene deconvolution on our bulk RNA sequencing data by using the “impute cell fractions” mode of CIBERSORTx. These analyses estimate the abundance of each cell type (described in the single-cell sequencing data) in our total RNAseq from liver tissues. Therefore, we can estimate the amount of healthy or scar-associated EC, quiescent or activated HSCs or quiescent or scar-associated macrophages. Therefore, the results of gene deconvolution showing an increase on these populations were considered to reflect endothelial capillarization, HSC activation and macrophage polarization associated to CLD progression. Note that we have renamed the following clusters from the original single-cell sequencing data [19] for clarity: capillarized_LSEC were originally defined as scar-associated ECs, and activated HSCs were originally defined as myofibroblast-like cells.

### Statistical analysis

Results were analysed with GraphPad Prism v8.0.2 (GraphPad Software). Data in the figures are expressed as mean ± standard error of the mean (S.E.M.). Differences among groups were tested for statistical significance by Student’s t-test. Differences were considered significant at p < 0.05.

## Results

### Portal hypertension in rats with ACLD

As expected, animals undergoing both cirrhosis models presented PH. TAA males exhibited a similar PP compared to females in the same model, while BDL males showed significantly higher PP than BDL females (+ 12%, p < 0.001) (Table 1). No differences were observed in PBF or IHVR. On the other hand, healthy females displayed a significantly higher spleen weight-to-body ratio than healthy males (+ 40%, p < 0.005). This difference was attenuated in the TAA model and reversed in the BDL model (-− 26%, p = 0.001), suggesting that males are more affected by splenomegaly associated with PH.

**Table 1:** Hepatic and systemic hemodynamic parameters in rats with advanced chronic liver disease

Parameter	Male control n = 11	Female control n = 11	p-value	Male TAA n = 18	Female TAA n = 18	p-value	Male BDL n = 12	Female BDL n = 12	p-value
MAP (mmHg)	81.3 ± 1.42	80.25 ± 2.13	> 0.2	83.1 ± 1.79	84.84 ± 2.27	> 0.2	80.18 ± 2.85	84.9 ± 4.06	> 0.2
HR (beats·min^−1^)	333.7 ± 6.91	344.5 ± 10.34	> 0.2	335.8 ± 10.48	345.5 ± 8.23	> 0.2	346.4 ± 8.96	360 ± 11.03	> 0.2
PP (mmHg)	8.46 ± 0.35	8.80 ± 0.24	> 0.2	14.25 ± 0.42	14.11 ± 0.47	> 0.2	14.24 ± 0.37	12.46 ± 0.38	0.006
PBF (mL·min^−1^·g^−1^)	2.24 ± 0.18	2.86 ± 0.21	0.03	1.22 ± 0.08	1.43 ± 0.13	0.17	0.74 ± 0.08	0.76 ± 0.09	> 0.2
HVR (mmHg· min^−1^·g^−1^)	3.96 ± 0.29	3.21 ± 0.22	0.04	12.48 ± 0.85	11.42 ± 1.24	> 0.2	21.8 ± 2.06	19.24 ± 2.88	> 0.2
Body weight (g)	417.1 ± 17.49	266.2 ± 4.37	< 0.0001	403.1 ± 7.42	278.1 ± 4.06	< 0.0001	428.5 ± 10.44	287.7 ± 6.29	< 0.0001
Liver weight ratio (%)	2.70 ± 0.07	2.85 ± 0.11	> 0.2	3.62 ± 0.06	3.76 ± 0.11	> 0.2	6.32 ± 0.29	6.75 ± 0.43	> 0.2
Spleen weight ratio (%)	0.19 ± 0.02	0.27 ± 0.01	0.005	0.34 ± 0.02	0.32 ± 0.02	> 0.2	0.57 ± 0.03	0.42 ± 0.03	0.001
Ascites (%)	0	0	NP	72	56	> 0.2	0	0	NP

Results are expressed as mean ± SEM; p values correspond to Student's t test. *HR* heart rate, *HVR* hepatic vascular resistance, *MAP* mean arterial pressure, *PBF* portal blood flow, *PP* portal pressure

### Sex-related differences in hepatic microvascular function and liver fibrosis

**Fig. 1:**
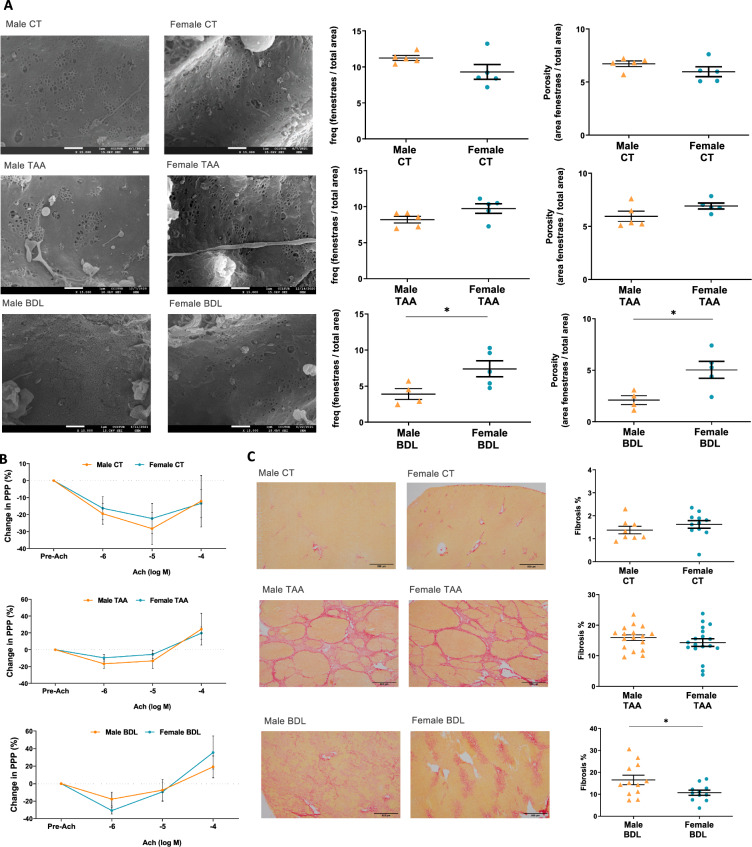
Sex-related differences in hepatic microvascular function and liver fibrosis. **A** Representative scanning electron microscopy images of hepatic sinusoidal areas (scale bar: 1µm), and corresponding quantification. **B** Hepatic microvascular function, calculated as the decrease in portal pressure in response to increasing doses of the endothelium-dependent vasodilator acetylcholine after vasoconstriction with methoxamine. **C** Sirius red staining of liver tissue sections (scale bar: 500 µm), and quantification. *p < 0.05 vs. male [Student’s t test for (**A**, **C**); repeated measures GLM for (**B**)]. N = 5 per group (**A**), n = 7 per group (**B**) and n = 11–18 per group (**C**). PPP, perfusion portal pressure

Liver sinusoids were analyzed by scanning electron microscopy, revealing that male control rats showed a tendency to have more fenestrae (+ 17%, p = 0.110) and higher porosity (+ 11%, p = 0.199) compared to female control rats. In disease, females showed a less aggressive phenotype, with more fenestrae (+ 19%, p = 0.095) and higher porosity (+ 16%, p = 0.12) compared to males in the TAA model. Similarly, in the BDL model, a significantly higher number of fenestrae (+ 89%, p = 0.043) and porosity (+ 140%, p = 0.022) was observed in the female cohort. These data suggest that males experience a more assertive disease development than females at a sinusoidal level (Fig. 1A). Nevertheless, hepatic microvascular function characterised through ex vivo liver perfusion experiments revealed no sex-related differences (Fig. 1B). Finally, staining of liver sections with Sirius red showed higher liver fibrosis in BDL males compared to females (− 35%, p = 0.03), with no sex-related differences in TAA or healthy rats (Fig. 1C).

### Sex differences in hepatic and renal function

To evaluate possible sex differences in liver enzymes, serum samples from TAA and BDL groups were analysed. AST and ALT levels were similar in male and female animals in the TAA group, whereas ALT levels tended to be higher in male BDL animals (+ 40%, p = 0.141) compared to females. Male rats in the TAA group exhibited a tendency toward lower levels of GGT (− 35%, p = 0.089) and higher levels of albumin (+ 10%, p = 0.068) compared to females. In the BDL group, males showed a significant increase in GGT levels (+ 61%, p < 0.001) and a tendency toward lower levels of urea (− 16%, p = 0.157) compared to females. Notably, serum bilirubin levels were significantly lower in BDL females compared to BDL males (− 30%, p < 0.001) (Supplementary Table 2).

### Transcriptomic analysis and sexual dimorphism in ACLD

To gain deeper insight into the differences in gene expression between males and females and to determine the molecular pathways and specific genes differentially regulated by sex, we performed bulk RNA sequencing on liver tissue from control, TAA and BDL groups.

The PCA revealed distinct clustering patterns among the different study groups. Samples from each group were clearly separated along the first two principal components, which together explained 63.5% of the total variance (PC1: 42.5%, PC2: 21.0%). The PCA plot showed a clear differentiation between the control, TAA, and BDL groups, suggesting significant divergence in gene expression profiles among these conditions. Additionally, within each experimental group, male and female samples were closely grouped, suggesting consistent sex-based patterns within the same condition (Supplementary Fig. 1).

**Fig. 2:**
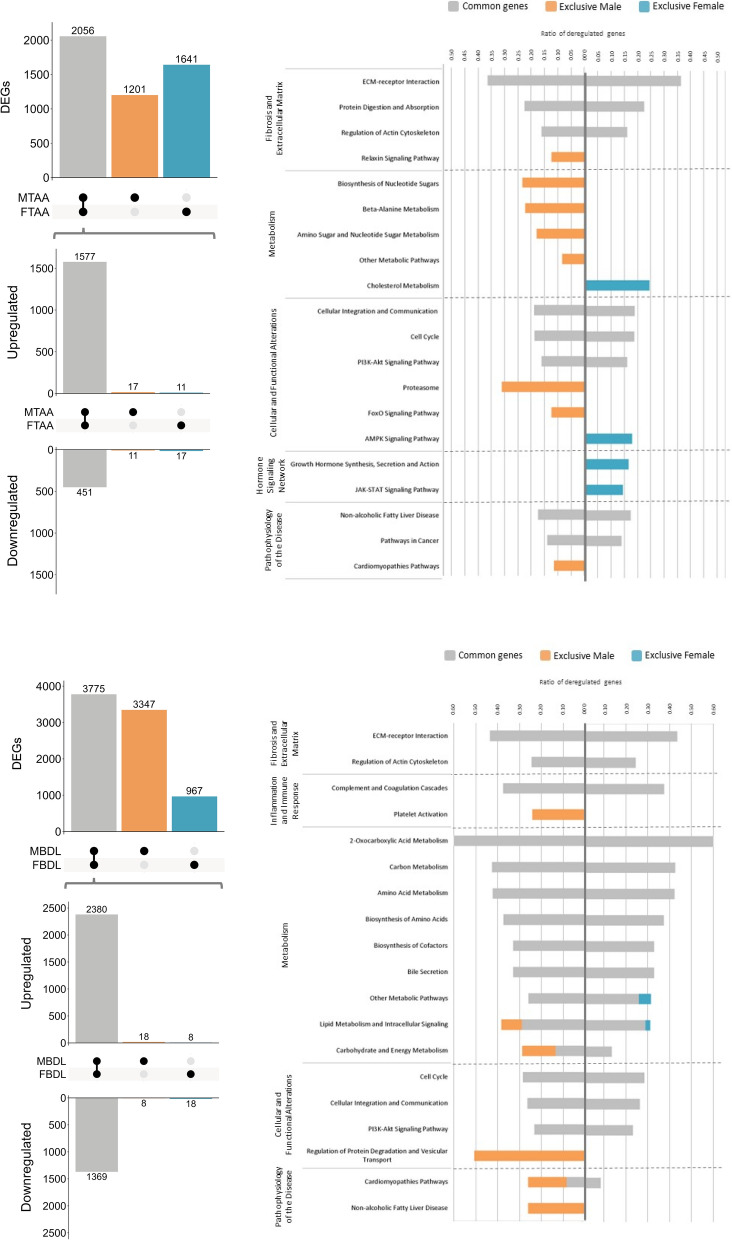
Transcriptomic analysis and sexual dimorphism in rats with advanced chronic liver disease. On the left, an upset plot of differentially expressed genes in TAA (top) and BDL (bottom) rats is shown. The number of differentially up- and downregulated genes for each sex is indicated in brackets, highlighting genes with sexual dimorphism. On the right, a bar plot displays the ratio of deregulated genes across various biological pathways using gPROFILER. Grey bars represent genes commonly deregulated in both sexes, orange bars represent genes exclusively deregulated in males, and blue bars represent genes exclusively deregulated in females. *MTAA* male TAA, *FTAA* female TAA, *MBDL* male BDL, *FBDL* female BDL

In TAA rats, transcriptomic analysis revealed similar dysregulation in diseased livers across both sexes, with 3257 and 3697 differentially expressed genes compared to healthy rats. Of those genes, 2056 were commonly dysregulated in both sexes, and were mainly associated with fibrosis and extracellular matrix. However, 1201 genes were exclusively dysregulated in males, mainly related to metabolism, while 1641 genes were exclusively dysregulated in females, linked to the hormone signalling network. In the BDL model, males exhibited greater dysregulation with 7122 versus 4742 differentially expressed genes. Most of those were common to both sexes, 3775, mainly associated with metabolism. Additionally, the nature of the affected pathways showed slight differences in the male group, with exclusive pathways (3347 versus 967) involving platelet activation, regulation of protein degradation, vesicular transport, and disease-related pathways (Fig. 2).

**Fig. 3:**
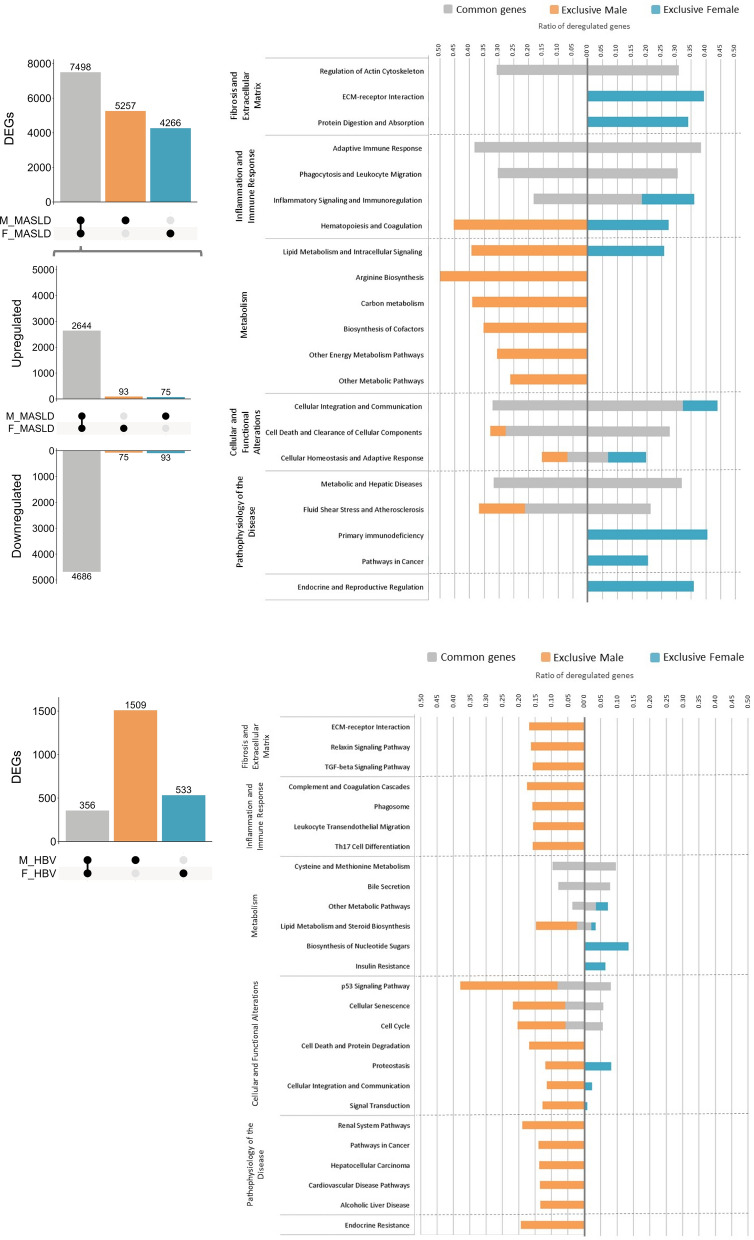
Transcriptomic analysis and sexual dimorphism in humans. On the left, an upset plot of differentially expressed genes in MASLD (top) and HBV (bottom) patients cohorts is shown. The number of differentially up- and downregulated genes for each sex is indicated in brackets, highlighting genes with sexual dimorphism. On the right, a bar plot displays the ratio of deregulated genes across various biological pathways using gPROFILER. Grey bars represent genes commonly deregulated in both sexes, orange bars represent genes exclusively deregulated in males, and blue bars represent genes exclusively deregulated in females. *M_MASLD* Male MASLD, *F_MASLD* female MASLD, *M_HBV* male HBV, *F_HBV* female HBV

Furthermore, we explored two well-defined cohorts of patients to identify potential similarities with preclinical models, aiming to provide translational perspectives. Transcriptomic analysis revealed comparable dysregulation patterns in MASLD livers across both sexes, with 12,755 and 11,764 differentially expressed genes compared to healthy individuals. Of these, 7498 genes were commonly dysregulated in both sexes, mainly associated with inflammation and immune response. However, 5257 genes were exclusively dysregulated in men, mostly related to metabolism, whereas 4266 genes were exclusively dysregulated in women, linked to fibrosis, extracellular matrix, and endocrine and reproductive regulation. In the HBV cohort, men exhibited a higher degree of dysregulation, 1865 versus 889 differentially expressed genes. Additionally, the pathways altered in men were more diverse, involving exclusive pathways (1509 versus 533) related to fibrosis and extracellular matrix, inflammation and immune response, cellular and functional alterations, and disease-related pathways (Fig. 3). The overall lower number of differentially expressed genes in the HBV cohort might be explained considering that HBV pathogenesis primarily involves immune-mediated liver damage rather than direct metabolic alterations, unlike MASLD, which is strongly associated with metabolic dysfunction.

Analysis of sexual dimorphism revealed 28 genes in TAA rats, 26 in BDL rats, and 168 in the MASLD cohort. These genes exhibit opposite expression patterns between sexes (Figs. 2 and 3). Many of these genes are suggested to play a significant role in ACLD, primarily related to metabolic processes, inflammation, and xenobiotic detoxification (Supplementary Table 3).

On the other hand, transcriptomic analysis revealed that male TAA, BDL, and MASLD groups exhibited numerous dysregulated pathways related to neurodegenerative diseases, whereas no such pathways were observed in females. Many of the genes involved are related to mitochondrial dysfunction and oxidative stress. This may suggest the existence of a liver-brain axis linked mainly to the male sex, in which the liver plays a key role in neuroinflammatory processes (Supplementary Fig. 2).

To investigate the potential protective role of oestrogens in female groups, we performed two complementary analyses. Serum levels of key sex hormones including oestradiol (E2), progesterone (Pg), and luteinizing hormone (LH) were measured using specific ELISA kits and revealed that BDL females exhibited higher levels of E2 and LH than healthy and a trend in comparison to TAA (E2 + 14%; p = 0.089), (Pg + 27%; p = 0.065) and (LH + 25%; p = 0.064) (Supplementary Fig. 3). In addition, we carried out GSEA using whole liver RNA-sequencing data from female animals in both the TAA and BDL cirrhosis models. In BDL females, we found significant negative enrichment of the bile secretion and primary bile acid biosynthesis pathways, consistent with cholestasis-induced impairment of bile flow. These alterations were supported by the downregulation of key transporter genes, including *Abcc2* (MRP2) and *Slc10a1* (NTCP). BDL females also showed significant positive enrichment of the oestrogen signalling pathway, which was not observed in TAA females. This was accompanied by the downregulation of genes involved in oestrogen inactivation, including *Sult1e1*, *Ugt2a3*, and *Cyp1a2*, suggesting impaired oestrogen clearance. In contrast, TAA females exhibited upregulation of *Sult1e1*, *Ugt2a3*, and *Cyp1a1*, consistent with preserved oestrogen metabolism and lack of oestrogen pathway activation. In addition, enrichment of the *cGMP-PKG *signalling and fluid shear stress and atherosclerosis pathways was detected exclusively in BDL females. Both pathways are associated with nitric oxide-mediated vasodilation, supporting a potential vasoprotective role of accumulated oestrogens in this model (Supplementary Fig. 4).

### Sex-dependent changes in hepatic cell subpopulations revealed by gene deconvolution

**Fig. 4:**
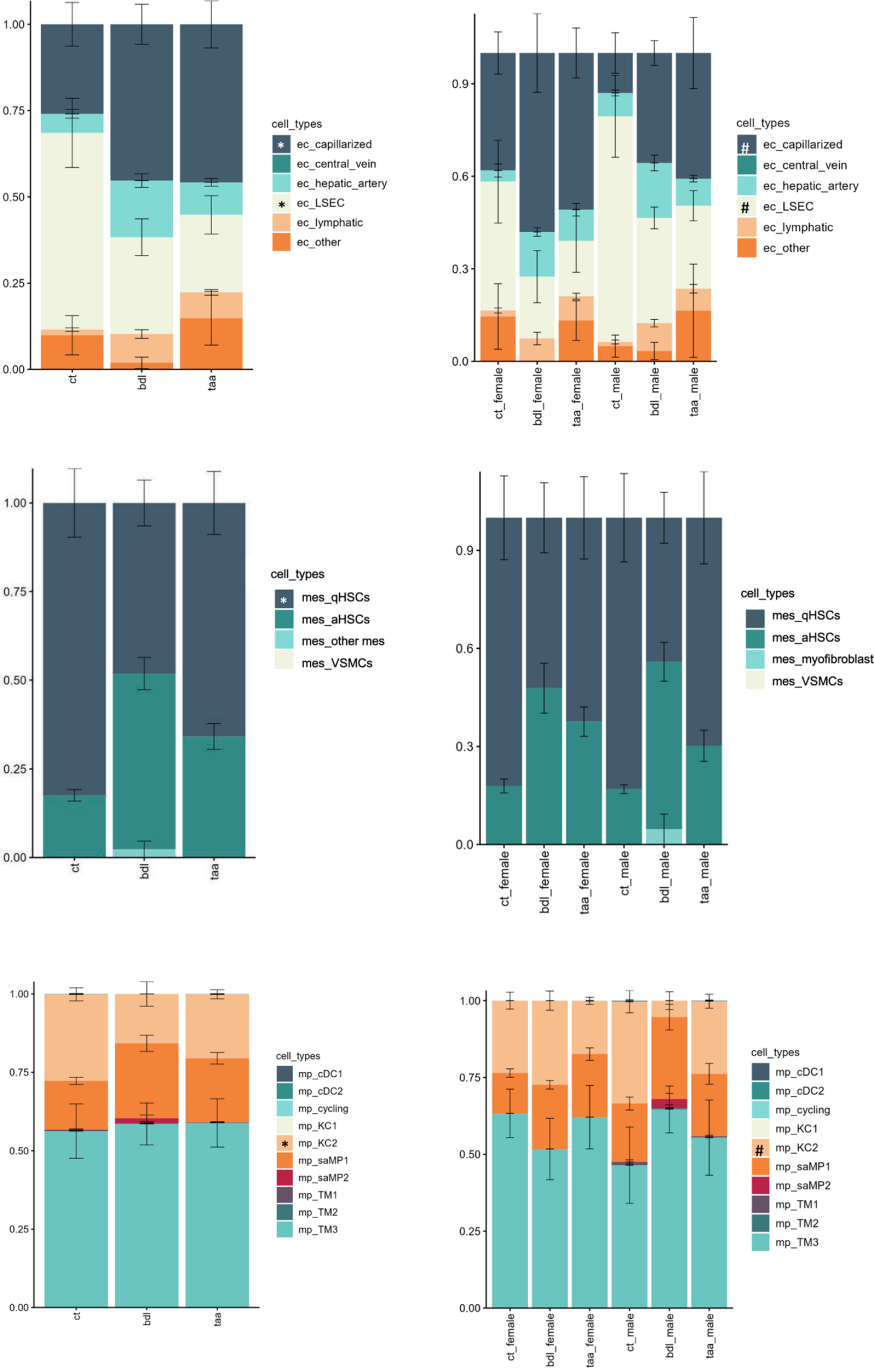
Sex-dependent changes in hepatic cell subpopulations in rats. Distribution of endothelial (top), mesenchymal (middle) and macrophage (bottom) cell type subpopulations in rats using gene deconvolution. On the left, CT, TAA, and BDL groups are compared. On the right, different sexes within each condition are compared. Single-cell sequencing data from Carlson et al. were used for the generation of the signature matrix. *p < 0.05, CT vs. disease. #p < 0.05, between sexes

Gene deconvolution analyses estimate the relative abundances of each sinusoidal cell type (including healthy or diseased phenotypes) in our total RNAseq transcriptome from liver tissues. Therefore, using these bioinformatic analyses we can observe changes in sinusoidal populations related to CLD. Specifically, the deconvolution data in Fig. 4 revealed profound changes in hepatic cell subpopulations in the livers of both animal models (left panels). Indeed, TAA and BDL animals showed more capillarized endothelial cells and a reduction of the healthy LSEC population compared to controls. These endothelial changes were accompanied by changes in the HSCs fractions, which showed a reduction of quiescent HSCs in favour of their activated phenotype, changes in the macrophage population, as seen by greater amounts of fibrosis-associated macrophages with a reduced proportion of healthy KCs. However, we observed further differences in sinusoidal cell subpopulations due to sex in both control and disease conditions (right panels). Along with the previously described differences in sinusoidal capillarization, deconvolution revealed that male rats exhibited a more specialized endothelial phenotype (LSEC) than females under control conditions, undergoing greater phenotype loss (higher abundance of capillarized ECs) during the disease. While no sex-related differences were observed in the HSC populations, males displayed a greater change in the macrophage population than females in the BDL group (greater reduction of KCs and increase in scar-associated macrophages).

**Fig. 5:**
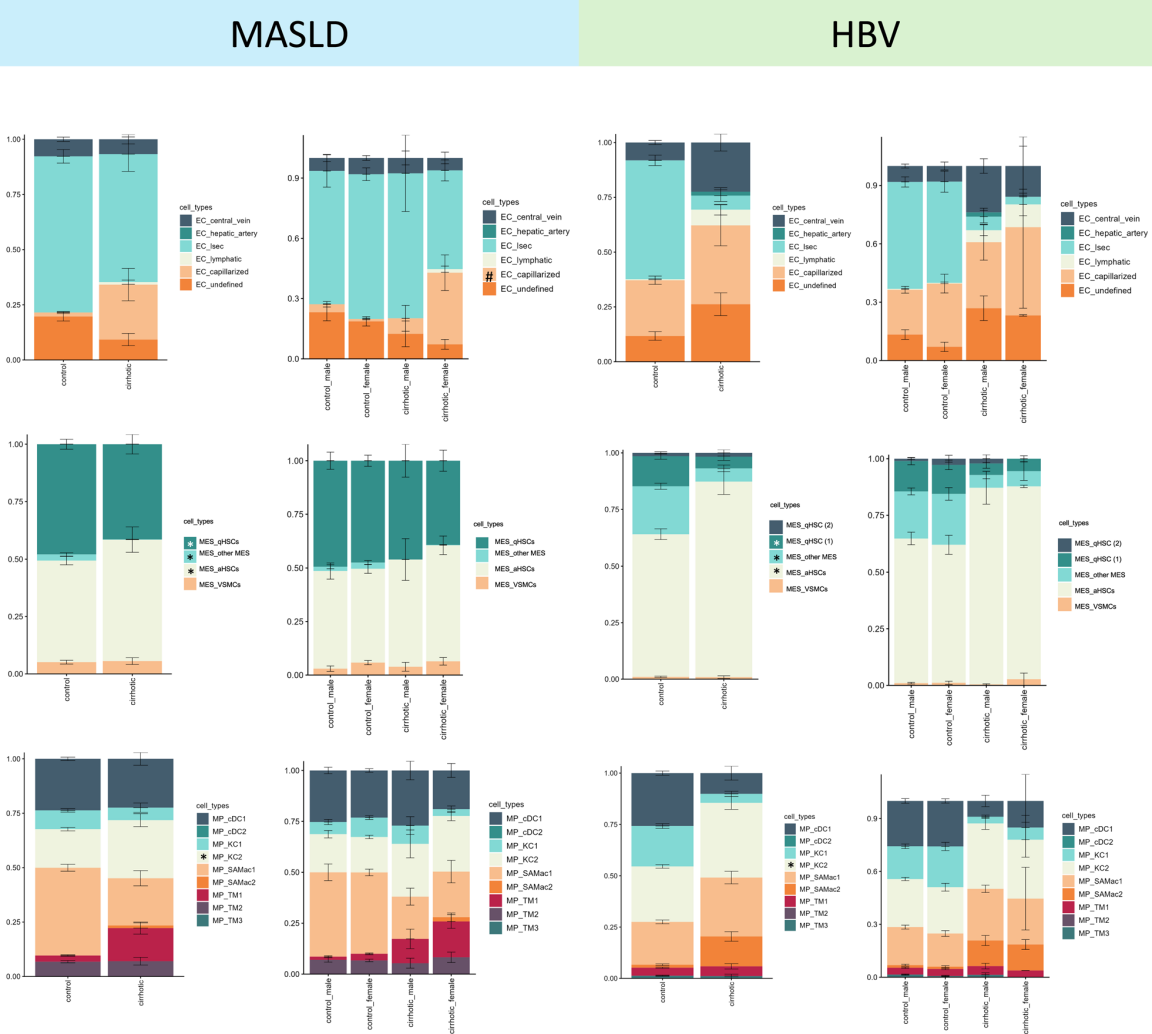
Sex-dependent changes in hepatic cell subpopulations in humans with liver disease. Distribution of endothelial (top), mesenchymal (middle) and macrophage (bottom) cell type subpopulations in MASLD and HBV human cohorts using gene deconvolution. On the left, control and disease groups are compared. On the right, different sexes within each condition are compared. Single-cell sequencing data from Ramachandran P et al. were used for the generation of the signature matrix. *p < 0.05, CT vs. disease. #p < 0.05, between sexes

In both human cohorts (Fig. 5), cirrhotic patients displayed loss of endothelial phenotype and increased macrophage and HSC activation compared to non-cirrhotic individuals (control vs cirrhotic panels). In the HBV cohort, we did not observe changes in sinusoidal cell abundances dependent on sex. On the other hand, data from the MASLD cohort showed that females were more sensitive to phenotype dysregulation in the three sinusoidal cell types, as observed by significant change in the LSEC and capillarized LSEC fractions, and by additional changes in the activated HSCs and scar-associated macrophage fractions. These findings correlate with the gene data described above, showing more accentuated pathway heterogeneity due to sex in the MASLD cohort than in the HBV cohort.

## Discussion

Chronic liver disease represents a major burden of disease worldwide, with cirrhosis being one of its most critical terminal manifestations. Despite advances in its pathophysiology understanding, the translation of preclinical findings into clinical practice continues to face significant barriers, one of which being the pharmacological and pathological discrepancy between sexes [21].

Traditionally, preclinical research in liver disease has prioritized the use of male rats over females, due to the perception that female hormonal variability could introduce a confounding factor in the interpretation of results. However, this practice has resulted in a knowledge gap on pathogenesis and treatment response based on sex, with implications for the occurrence of adverse effects and lack of therapeutic efficacy once drugs are administered to the general population, which includes both sexes [5, 22], thus representing a key limiting factor in the development of effective and safe therapeutic strategies.

Although some studies have investigated sex-related differences in liver disease, the available evidence remains limited and is mostly restricted to metabolic models like steatosis, where females often present with milder disease [23]. In contrast, advanced models such as BDL or hepatotoxin-induced cirrhosis have been much less explored. As a result, we still lack a clear understanding of how sex affect disease severity and main complications, including portal hypertension, in these contexts.

As demonstrated in our project, sex significantly influences pathophysiology and underlying mechanisms of CLD. In the TAA model, a similar degree of disease is achieved in both sexes, with no significant differences in PH, sinusoidal phenotype, fibrosis or liver function. However, important sex-related differences were observed in the transcriptomic analysis. Males exhibited dysregulated pathways involved in metabolism, while females showed deregulation in hormone signalling genes.

Previous studies on TAA-induced cirrhosis in male rats have shown beneficial effects on PH using drugs like obeticholic acid, a potent and selective FXR agonist involved in bile acid and lipid metabolism, and liraglutide, a glucagon-like peptide-1 receptor agonist used in diabetes treatment [24]. Notably, liraglutide also demonstrated antifibrotic effects by reducing hepatic inflammation, likely through modulation of metabolic pathways and improvement of insulin resistance [25]. Given the metabolic dysregulation observed in male rats, further investigation into the effects of these drugs in female rats with TAA-induced cirrhosis could provide critical insights into potential therapeutic mechanisms. Additionally, when considering cell populations, treatment effects could be different for example on LSECs, since male rats displayed a more specialized endothelial phenotype than females under control conditions, leading to a greater degree of capillarized-like endothelia during the disease.

Additionally, the BDL model revealed a pronounced sex difference in disease severity, emphasizing the importance of sex-specific research. Specifically, females develop a significantly milder form compared to males, with notable differences such as reduced PH, increased LSEC fenestration, and a lower degree of fibrosis. Consistently, bilirubin levels were significantly lower in BDL females, further indicating reduced cholestatic injury and better-preserved liver function in this group. This difference in disease severity between sexes may be attributed to several factors, including the protective role of oestrogens in females [26, 27] and the sexually dimorphic nature of the organ [11]. Indeed, our observations align with previous evidence showing that oestrogens have a range of protective effects on hepatocytes, including reducing susceptibility to steatosis, increasing mitochondrial content and oxidative capacity, and thereby decreasing lipid accumulation and oxidative stress [28, 29]. Therefore, perhaps the duration of disease (28 days) and oestrogen protection in the female may have prevented as much liver injury and fibrosis compared to the severity in the male.

In support of this hypothesis, our GSEA-derived analyses and biochemical data indicate that BDL females exhibit active oestrogen signalling, as evidenced by pathway enrichment, elevated serum oestradiol levels and lower expression of oestrogen-inactivating enzymes. This probably leads to a functional accumulation of oestrogen, contributing to vascular protection. Consistently, BDL females showed increased porosity and fenestration in hepatic sinusoids compared to BDL males. In contrast, the absence of similar regulatory features in TAA females may explain the lack of sex-related differences at the molecular level. Overall, these findings suggest that oestrogen accumulation in BDL females prevents severe disease progression and plays a central role in the sexually dimorphic response to cirrhosis and portal hypertension.

Recognizing this sex-specific modulation is especially relevant when considering therapeutic strategies, as the severity and liver distortion caused by the disease will likely require a different approach. Although previous studies have reported beneficial effects of Droxidopa [30], recombinant human manganese superoxide dismutase (rMnSOD) [31], and obeticholic acid, as mentioned earlier [24], in the BDL model, these investigations were only developed in male rats. Moreover, taking into account differences in sub-cellular populations, treatments’ effects might be more significant in males, as they exhibited greater change in macrophage and LSEC populations than females in the BDL group. For example, our group showed that rMnSOD significantly improves the LSEC phenotype [31], while Steib et al. suggested that Kupffer cell activation plays a crucial role in increasing portal pressure through the production of vasoconstrictive agents like thromboxane A2 [32]. Both studies were conducted in BDL male rats, thus it would be very interesting to explore how these cell types would behave under similar conditions in females.

As stated before, and despite the inclusion of both sexes in clinical trials for liver diseases, preclinical models often fail to account for sex differences, which may contribute to the failure of many drugs that initially showed promise in these models. For instance, a possible reason for recent failures in clinical trials evaluating anti-fibrotics for steatotic liver disease [33–35] could be the lack of preclinical data of these antifibrotic drugs in females. In fact, our transcriptomic data revealed distinct deregulation of pathways related to fibrosis and the extracellular matrix in females with MASLD in comparison to male. Additionally, it is important to reiterate the potential role of sex hormones in women, particularly since their regulation is often disrupted in chronic diseases [36]. Specifically, women with cirrhosis who are also menopausal exhibit more severe disease compared to premenopausal women [27, 37, 38], which is herein reflected dysregulation of related transcriptional pathways. Furthermore, considering the significant attention being directed towards the development of treatments for MASH, assessment of novel drugs should also consider the sub-cellular transcriptomic heterogeneity due to sex observed in this study, where male patients exhibited different phenotypic deregulations compared to female.

Sexual dimorphism and hormonal influences are critical considerations in the development of therapeutic strategies, as dysregulation of hormonal signalling pathways has been observed in transcriptional processes. In the mammalian liver, sexual dimorphism is largely driven by the sex-specific secretion patterns of growth hormone, which is pulsatile in males and more continuous in females, significantly impacting liver physiology by regulating regeneration, cell proliferation, and metabolism. Recognizing sex differences in gene expression, body composition, and metabolic processes, including inflammation and xenobiotic detoxification, is essential for personalized medicine [36, 39]. This understanding will inform the design of more targeted drugs, considering pharmacokinetic and pharmacodynamic differences between sexes [40, 41].

The limitations of this study highlight the need for further research, particularly regarding age and hormonal variations. One significant limitation is that our study was performed in young rats, which may not fully represent the effects of aging on the observed changes due to hormonal variations [42, 43]. Future studies should include aged rats to determine whether these changes are exacerbated or mitigated over time. In addition, it would be valuable to study pregnant [44] or ovariectomized females [37], as well as to monitor the oestrous cycle of female rats [45] or testosterone levels in male rats [46], which was not accounted for in this study. Although ovariectomy is commonly used to model oestrogen deficiency, it induces systemic metabolic changes that may complicate proper interpretation of results [47]. For this reason, we prioritized studying intact females to better reflect physiological conditions. Furthermore, it is important to recognize that no preclinical model can perfectly mimic human disease, especially in the context of MASLD, given the complexity and multifactorial nature of its aetiology. This complexity makes it difficult to identify specific genes for translational research [48].

On the other hand, the main strengths of our study include the validation of animal data in human samples and the use of bioinformatic techniques for the evaluation of cellular subpopulations in humans. Indeed, the use of gene deconvolution on human samples has been proposed before as a realistic alternative to single-cell sequencing for the study of hepatic cell subpopulations [49]. These analyses offer the possibility to study changes in sinusoidal cell frequencies associated with the progression of CLD in whole tissue samples, without the need to isolate individual cell types. Indeed, our results from gene deconvolution in humans provide further mechanistic insight of some of the molecular pathway dimorphisms shown in Fig. 3. For example, our data indicate that women display alterations in pathways related to fibrosis and inflammation, which correlate with greater differences in sinusoidal alterations observed in the gene deconvolution analyses. On the other hand, men show greater metabolic alterations than women, which is probably related to hepatocyte function and less sinusoidal cell-related, as also indicated by fewer changes in gene deconvolution. Therefore, the use of these bioinformatic techniques allowed the interpretation of whole transcriptome analyses at the sinusoidal level.

### Perspectives and significance

This study provides critical insights into the sex-specific mechanisms underlying chronic liver disease and portal hypertension, highlighting significant molecular and cellular differences between males and females in both preclinical models and human cohorts. These findings underscore the necessity of considering sex as a biological variable in liver disease research, particularly for tailoring therapeutic strategies. The identification of distinct transcriptomic patterns and cellular responses opens avenues for the development of sex-specific biomarkers and targeted interventions that could improve outcomes for both sexes. This study not only seeks to fill a gap in the scientific literature but also advocates for a necessary revision of the current paradigms in preclinical research, promoting an equitable inclusion of both sexes in the early phases of drug development. This is expected to reduce the incidence of adverse effects and improve the efficacy of treatments for chronic liver disease in both sexes. Expanding these findings to diverse experimental models and broader patient populations will help validate their translational significance and guide the development of more inclusive and tailored therapeutic strategies for managing liver disease.

## Conclusions

This study highlights sex-dependent molecular and cellular differences in the pathophysiology of cirrhosis and portal hypertension in two preclinical models. Male and female rats showed distinct phenotypes in disease severity, liver fibrosis and vascular remodelling, supported by transcriptomic analyses that revealed sex-specific molecular pathways, mainly with a greater difference in the BDL model. These findings were corroborated in patients, emphasising the translational relevance of the study. Further research in preclinical models and in diverse patient cohorts is essential to develop safe and effective treatments for advanced chronic liver disease that address the unique needs of both sexes.

## Supplementary Information

Supplementary Material 1.

## Data Availability

No datasets were generated or analysed during the current study.

## References

[1] Gracia-Sancho J, Marrone G, Fernández-Iglesias A. Hepatic microcirculation and mechanisms of portal hypertension. Nat Rev Gastroenterol Hepatol. 2019;16(4):221–34. 30568278 10.1038/s41575-018-0097-3

[2] Guixé-Muntet S, Quesada-Vázquez S, Gracia-Sancho J. Pathophysiology and therapeutic options for cirrhotic portal hypertension. Lancet Gastroenterol Hepatol. 2024;9(7):646–63. 38642564 10.1016/S2468-1253(23)00438-7

[3] Abad-Jorda L, Martínez-Alcocer A, Guixé-Muntet S, Hunt NJ, Westwood LJ, Lozano JJ, et al. miR-27b-3p modulates liver sinusoidal endothelium dedifferentiation in chronic liver disease. Hepatol Commun. 2025. 10.1097/HC9.0000000000000700. 40304581 10.1097/HC9.0000000000000700 PMC12045533

[4] Nevzorova YA, Boyer-Diaz Z, Cubero FJ, Gracia-Sancho J. Animal models for liver disease—a practical approach for translational research. J Hepatol. 2020;73(2):423–40. 32330604 10.1016/j.jhep.2020.04.011

[5] Shansky RM, Murphy AZ. Considering sex as a biological variable will require a global shift in science culture. Nat Neurosci. 2021;24(4):457–64. 33649507 10.1038/s41593-021-00806-8 PMC12900283

[6] Florido A, Velasco ER, Soto-Faguás CM, Gomez-Gomez A, Perez-Caballero L, Molina P, et al. Sex differences in fear memory consolidation via Tac2 signaling in mice. Nat Commun. 2021;12(1):2496. 33941789 10.1038/s41467-021-22911-9 PMC8093426

[7] Sacramento JF, Martins FO, Rodrigues T, Matafome P, Ribeiro MJ, Olea E, et al. A2 adenosine receptors mediate whole-body insulin sensitivity in a prediabetes animal model: primary effects on skeletal muscle. Front Endocrinol (Lausanne). 2020;11(April):1–16. 32411098 10.3389/fendo.2020.00262 PMC7198774

[8] Shansky RM. Are hormones a “female problem” for animal research? Science. 2019;364(6443):825–6. 31147505 10.1126/science.aaw7570

[9] Liu Y, Chen Y, Xie X, Yin A, Yin Y, Liu Y, et al. Gender difference on the effect of omega-3 polyunsaturated fatty acids on acetaminophen-induced acute liver failure. Oxid Med Cell Longev. 2020;2020:8096847. 32908639 10.1155/2020/8096847 PMC7474378

[10] Yan H, Wu W, Chang X, Xia M, Ma S, Wang L, et al. Gender differences in the efficacy of pioglitazone treatment in nonalcoholic fatty liver disease patients with abnormal glucose metabolism. Biol Sex Differ. 2021;12(1):1–8. 33397443 10.1186/s13293-020-00344-1 PMC7784274

[11] Buzzetti E, Parikh PM, Gerussi A, Tsochatzis E. Gender differences in liver disease and the drug-dose gender gap. Pharmacol Res. 2017;120:97–108. 28336373 10.1016/j.phrs.2017.03.014

[12] Selicean SE, Felli E, Wang C, Nulan Y, Lozano JJ, Guixé-Muntet S, et al. Stiffness-induced modulation of ERG transcription factor in chronic liver disease. NPJ Gut Liver. 2024;1(1):7. 39381160 10.1038/s44355-024-00007-7 PMC11459910

[13] Boyer-Diaz Z, Domingo JC, de Gregorio E, Manicardi N, Aristu-Zabalza P, Cordobilla B, et al. A nutraceutical rich in docosahexaenoic acid improves portal hypertension in a preclinical model of advanced chronic liver disease. Nutrients. 2019;11(10). 10.3390/nu11102358 PMC6835927 31623374

[14] Boyer-Diaz Z, Aristu-Zabalza P, Andrés-Rozas M, Robert C, Ortega-Ribera M, Fernández-Iglesias A, et al. Pan-PPAR agonist lanifibranor improves portal hypertension and hepatic fibrosis in experimental advanced chronic liver disease. J Hepatol. 2021;74(5):1188–99. 33278455 10.1016/j.jhep.2020.11.045

[15] Gupta TK, Toruner M, Chung MK, Groszmann RJ. Endothelial dysfunction and decreased production of nitric oxide in the intrahepatic microcirculation of cirrhotic rats. Hepatology. 1998;28(4I):926–31. 9755227 10.1002/hep.510280405

[16] Kolberg L, Raudvere U, Kuzmin I, Adler P, Vilo J, Peterson H. G:Profiler-interoperable web service for functional enrichment analysis and gene identifier mapping (2023 update). Nucleic Acids Res. 2023;51(W1):W207–12. 37144459 10.1093/nar/gkad347 PMC10320099

[17] The GTEx Consortium atlas of genetic regulatory effects across human tissues. Science. 2020;369(6509):1318–30. 10.1126/science.aaz1776 PMC7737656 32913098

[18] Carlson KN, Verhagen JC, Jennings H, Verhoven B, McMorrow S, Pavan-Guimaraes J, et al. Single-cell RNA sequencing distinguishes dendritic cell subsets in the rat, allowing advanced characterization of the effects of FMS-like tyrosine kinase 3 ligand. Scand J Immunol. 2022;96(1): e13159. 35285040 10.1111/sji.13159 PMC9250598

[19] Ramachandran P, Dobie R, Wilson-Kanamori JR, Dora EF, Henderson BEP, Luu NT, et al. Resolving the fibrotic niche of human liver cirrhosis at single-cell level. Nature. 2019;575(7783):512–8. 31597160 10.1038/s41586-019-1631-3 PMC6876711

[20] Newman AM, Steen CB, Liu CL, Gentles AJ, Chaudhuri AA, Scherer F, et al. Determining cell type abundance and expression from bulk tissues with digital cytometry. Nat Biotechnol. 2019;37(7):773–82. 31061481 10.1038/s41587-019-0114-2 PMC6610714

[21] Asrani SK, Devarbhavi H, Eaton J, Kamath PS. Burden of liver diseases in the world. J Hepatol. 2019;70(1):151–71. 30266282 10.1016/j.jhep.2018.09.014

[22] Mauvais-Jarvis F, Bairey Merz N, Barnes PJ, Brinton RD, Carrero JJ, DeMeo DL, et al. Sex and gender: modifiers of health, disease, and medicine. Lancet. 2020;396(10250):565–82. 32828189 10.1016/S0140-6736(20)31561-0 PMC7440877

[23] Lonardo A, Nascimbeni F, Ballestri S, Fairweather DL, Win S, Than TA, et al. Sex differences in nonalcoholic fatty liver disease: state of the art and identification of research gaps. Hepatology. 2019;70(4):1457–69. 30924946 10.1002/hep.30626 PMC6766425

[24] Verbeke L, Farre R, Trebicka J, Komuta M, Roskams T, Klein S, et al. Obeticholic acid, a farnesoid X receptor agonist, improves portal hypertension by two distinct pathways in cirrhotic rats. Hepatology. 2014;59(6):2286–98. 24259407 10.1002/hep.26939

[25] De Mesquita FC, Guixé-Muntet S, Fernández-Iglesias A, Maeso-DIáz R, Vila S, Hide D, et al. Liraglutide improves liver microvascular dysfunction in cirrhosis: evidence from translational studies. Sci Rep. 2017;7(1):1–10. 28607430 10.1038/s41598-017-02866-y PMC5468330

[26] Barouki R, Samson M, Blanc EB, Colombo M, Zucman-Rossi J, Lazaridis KN, et al. The exposome and liver disease—how environmental factors affect liver health. J Hepatol. 2023;79(2):492–505. 36889360 10.1016/j.jhep.2023.02.034 PMC10448911

[27] Burra P, Zanetto A, Schnabl B, Reiberger T, Montano-Loza AJ, Asselta R, et al. Hepatic immune regulation and sex disparities. Nat Rev Gastroenterol Hepatol. 2024;21(12):869-84. 39237606 10.1038/s41575-024-00974-5

[28] Cooper KM, Delk M, Devuni D, Sarkar M. Sex differences in chronic liver disease and benign liver lesions. JHEP Rep. 2023;5(11): 100870. 37791378 10.1016/j.jhepr.2023.100870 PMC10542645

[29] Xu L, Yuan Y, Che Z, Tan X, Wu B, Wang C, et al. The hepatoprotective and hepatotoxic roles of sex and sex-related hormones. Front Immunol. 2022;13(July):1–18. 10.3389/fimmu.2022.939631 PMC9289199 35860276

[30] Coll M, Rodriguez S, Raurell I, Ezkurdia N, Brull A, Augustin S, et al. Droxidopa, an oral norepinephrine precursor, improves hemodynamic and renal alterations of portal hypertensive rats. Hepatology. 2012;56(5):1849–60. 22610782 10.1002/hep.25845

[31] Guillaume M, Rodriguez-Vilarrupla A, Gracia-Sancho J, Rosado E, Mancini A, Bosch J, et al. Recombinant human manganese superoxide dismutase reduces liver fibrosis and portal pressure in CCl4-cirrhotic rats. J Hepatol. 2013;58(2):240–6. 22989570 10.1016/j.jhep.2012.09.010

[32] Steib CJ, Gerbes AL, Bystron M, op denWinkel M, Härtl J, Roggel F, et al. Kupffer cell activation in normal and fibrotic livers increases portal pressure via thromboxane A2. J Hepatol. 2007;47(2):228–38. 17573142 10.1016/j.jhep.2007.03.019

[33] Harrison SA, Abdelmalek MF, Caldwell S, Shiffman ML, Diehl AM, Ghalib R, et al. Simtuzumab is ineffective for patients with bridging fibrosis or compensated cirrhosis caused by nonalcoholic steatohepatitis. Gastroenterology. 2018;155(4):1140–53. 29990488 10.1053/j.gastro.2018.07.006

[34] Harrison SA, Wong VWS, Okanoue T, Bzowej N, Vuppalanchi R, Younes Z, et al. Selonsertib for patients with bridging fibrosis or compensated cirrhosis due to NASH: Results from randomized phase III STELLAR trials. J Hepatol. 2020;73(1):26–39. 32147362 10.1016/j.jhep.2020.02.027

[35] Anstee QM, Neuschwander-Tetri BA, Wai-Sun Wong V, Abdelmalek MF, Rodriguez-Araujo G, Landgren H, et al. Cenicriviroc lacked efficacy to treat liver fibrosis in nonalcoholic steatohepatitis: AURORA phase III randomized study. Clin Gastroenterol Hepatol. 2024;22(1):124-134.e1. 37061109 10.1016/j.cgh.2023.04.003

[36] Della TS. Beyond the x factor: relevance of sex hormones in nafld pathophysiology. Cells. 2021;10(9). 10.3390/cells10092502 PMC8470830 34572151

[37] Lee YH, Son JY, Kim KS, Park YJ, Kim HR, Park JH, et al. Estrogen deficiency potentiates thioacetamide-induced hepatic fibrosis in Sprague-Dawley rats. Int J Mol Sci. 2019;20(15):1–16. 10.3390/ijms20153709 PMC6696236 31362375

[38] Yang JD, Abdelmalek MF, Pang H, Guy CD, Smith AD, Diehl AM, et al. Gender and menopause impact severity of fibrosis among patients with nonalcoholic steatohepatitis. Hepatology. 2014;59(4):1406–14. 24123276 10.1002/hep.26761 PMC3966932

[39] Priego-Parra BA, Gallego-Durán R, Román-Calleja BM, Velarde-Ruiz Velasco JA, Romero-Gómez M, Gracia-Sancho J. Advancing precision medicine in metabolic dysfunction-associated steatotic liver disease. Trends Endocrinol Metab. 2025. 10.1016/j.tem.2025.03.006. 40221323 10.1016/j.tem.2025.03.006

[40] Waxman DJ, Holloway MG. Sex differences in the expression of hepatic drug metabolizing enzymes. Mol Pharmacol. 2009;76(2):215–28. 19483103 10.1124/mol.109.056705 PMC2713118

[41] Zucker I, Prendergast BJ. Sex differences in pharmacokinetics predict adverse drug reactions in women. Biol Sex Differ. 2020;11(1):1–14. 32503637 10.1186/s13293-020-00308-5 PMC7275616

[42] Ortega-Ribera M, Hunt NJ, Gracia-Sancho J, Cogger VC. The hepatic sinusoid in aging and disease: update and advances from the 20th liver sinusoid meeting. Hepatol Commun. 2020;4(7):1087–98. 32626839 10.1002/hep4.1517 PMC7327202

[43] Maeso-Díaz R, Ortega-Ribera M, Lafoz E, JoséLozano J, Baiges A, Francés R, et al. Aging influences hepatic microvascular biology and liver fibrosis in advanced chronic liver disease. Aging Dis. 2019;10(4):684–98. 31440376 10.14336/AD.2019.0127 PMC6675529

[44] Saad MJA, Maeda L, Brenelli SL, Carvalho CRO, Paiva RS, Velloso LA. Defects in insulin signal transduction in liver and muscle of pregnant rats. Diabetologia. 1997;40(2):179–86. 9049478 10.1007/s001250050660

[45] Lovick TA, Zangrossi H. Effect of estrous cycle on behavior of females in rodent tests of anxiety. Front Psychiatry. 2021;12(August):1–20. 10.3389/fpsyt.2021.711065 PMC8438218 34531768

[46] Machida T, Yonezawa Y, Noumura T. Age-associated changes in plasma testosterone levels in male mice and their relation to social dominance or subordinance. Horm Behav. 1981;15(3):238–45. 7298026 10.1016/0018-506x(81)90013-1

[47] Lei Z, Wu H, Yang Y, Hu Q, Lei Y, Liu W, et al. Ovariectomy impaired hepatic glucose and lipid homeostasis and altered the gut microbiota in mice with different diets. Front Endocrinol (Lausanne). 2021;12(June):1–19. 10.3389/fendo.2021.708838 PMC8278766 34276568

[48] Vacca M, Kamzolas I, Harder LM, Oakley F, Trautwein C, Hatting M, et al. An unbiased ranking of murine dietary models based on their proximity to human metabolic dysfunction-associated steatotic liver disease (MASLD). Nat Metab. 2024;6(6):1178–96. 38867022 10.1038/s42255-024-01043-6 PMC11199145

[49] Kim A, Wu X, Allende DS, Nagy LE. Gene deconvolution reveals aberrant liver regeneration and immune cell infiltration in alcohol-associated hepatitis. Hepatology. 2021;74(2):987–1002. 33619773 10.1002/hep.31759 PMC8475730

