# Supplementary material for: Sex-specific differences in preclinical models of advanced chronic liver disease and portal hypertension
Source: Biol Sex Differ. 2025 Jun 3;16:39. doi: 10.1186/s13293-025-00721-8 (PMC12131374; doi:10.1186/s13293-025-00721-8)
Supplement: Supplementary file 1 — Supplementary Material 1. [file 13293_2025_721_MOESM1_ESM.pdf]

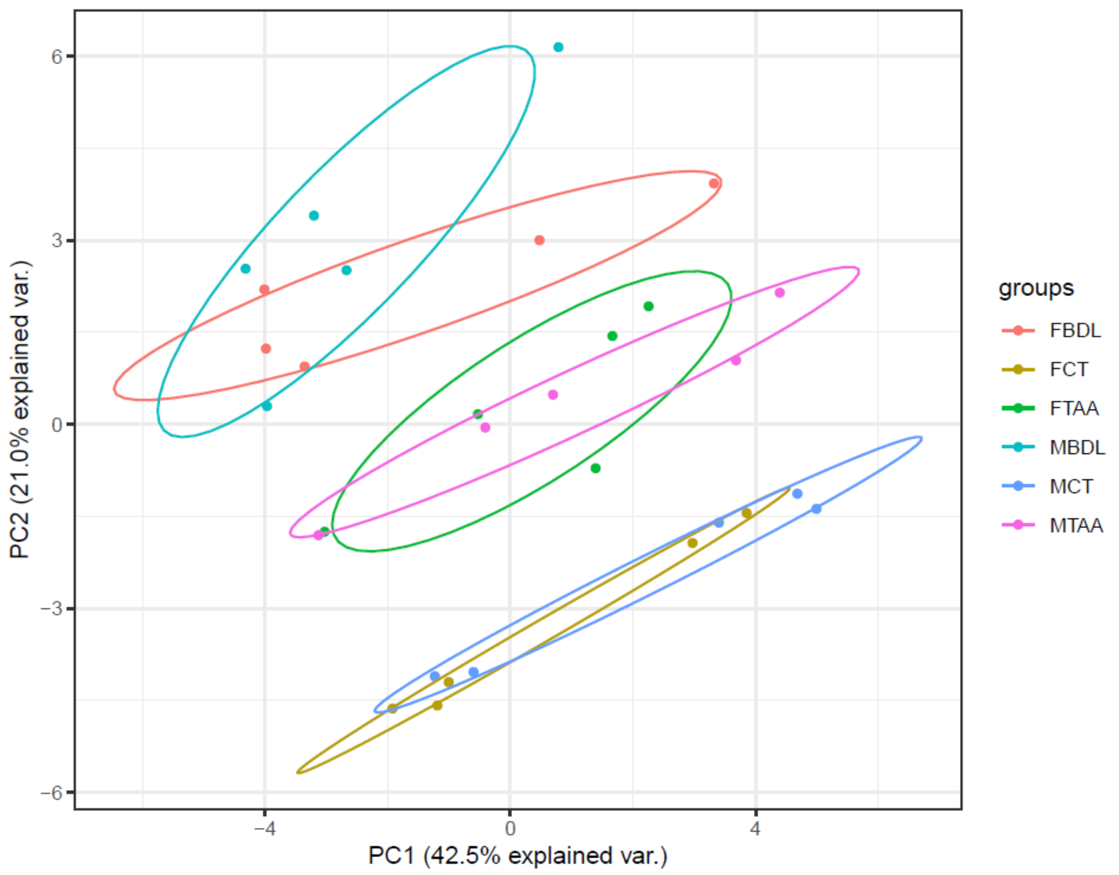

Supplementary figure 1. PCA reveals sex-based patterns in gene expression profiles. The PCA shows distinct clustering patterns between study groups along the first two principal components (PC1: 42.5%, PC2: 21.0%), explaining 63.5% of the total variance. Samples are grouped based on sex and condition. n = 5 per group. MCT: male CT; FCT: female CT; MTAA: male TAA; FTAA: female TAA; MBDL: male BDL; FBDL: female BDL.

TAA_MALE	Pathway	Genes	PValue
	Parkinson disease	42	7.06E-09
	Alzheimer disease	53	7.36E-09
	Prion disease	42	9.83E-09
	Amyotrophic lateral sclerosis	45	2.62E-06
	Huntington disease	39	5.06E-06
	Pathways of neurodegeneration - multiple diseases	51	1.97E-05
	Spinocerebellar ataxia	21	2.13E-04
	Retrograde endocannabinoid signaling	17	1.25E-02

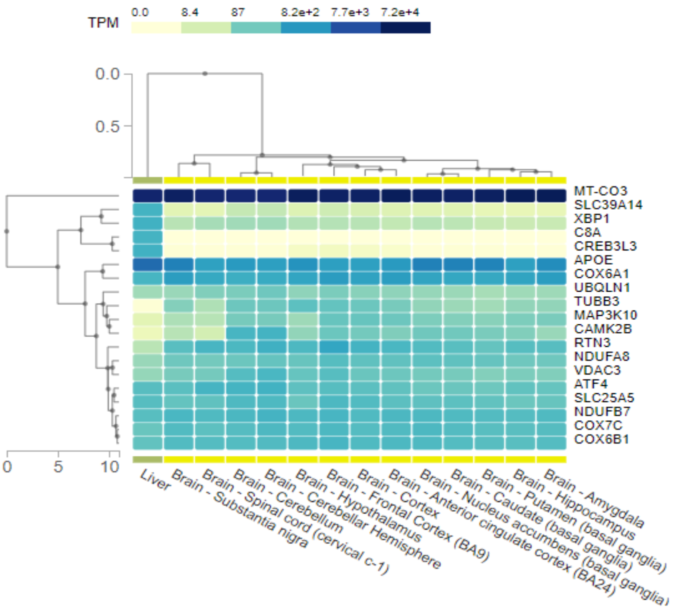

BDL_MALE	Pathway	Genes	PValue
	Parkinson disease	102	9.03E-21
	Huntington disease	109	8.94E-20
	Prion disease	96	2.70E-17
	Alzheimer disease	120	6.37E-16
	Amyotrophic lateral sclerosis	112	2.49E-14
	Spinocerebellar ataxia	45	1.22E-06
	Retrograde endocannabinoid signaling	42	5.37E-05
	Axon guidance	39	1.46E-02
	Dopaminergic synapse	28	4.34E-02

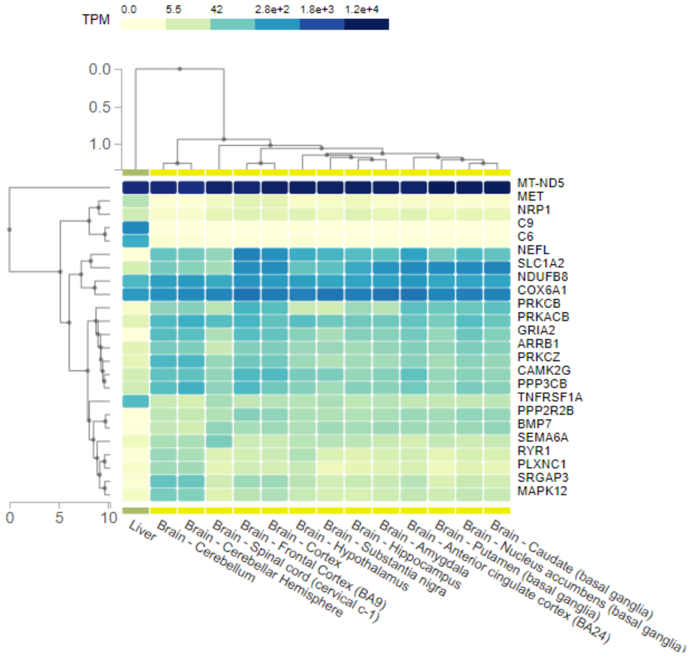

HUMAN MASLD_MALE	Pathway	Genes	PValue
	Parkinson disease	89	3.05E-09
	Huntington disease	95	7.01E-08
	Prion disease	82	4.75E-06
	Alzheimer disease	106	8.14E-06
	Pathways of neurodegeneration - multiple diseases	121	1.35E-04
	Amyotrophic lateral sclerosis	95	6.68E-04

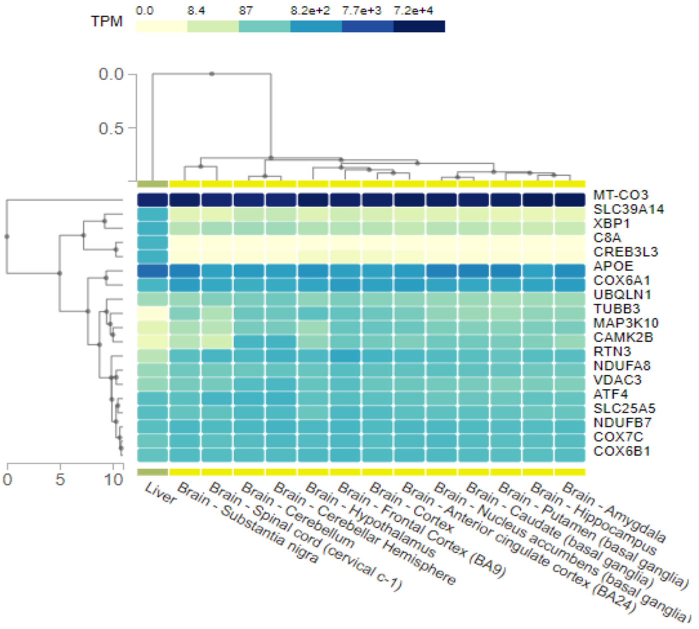

Supplementary figure 2. Dysregulated neurodegenerative pathways in males. On the left, transcriptomic analysis shows that male TAA, BDL, and human MASLD groups exhibited numerous dysregulated pathways related to neurodegenerative diseases, while no such pathways were observed in females. On the right, a selection of genes that may play an important role in neurodegenerative diseases is displayed. These genes were analyzed using the GTEx portal, which compares gene expression across various tissues.

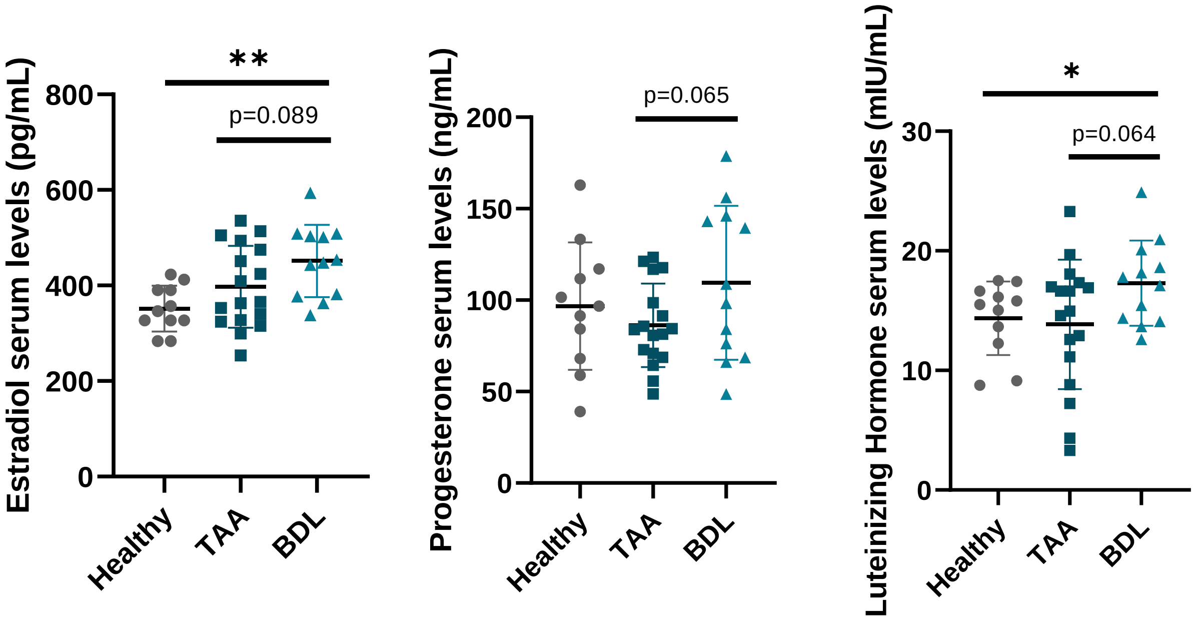

Supplementary figure 3. Measurement of sex hormones in serum. The figure summarizes serum levels of estradiol, progesterone, and luteinizing hormone (LH) in healthy, TAA, and BDL female rats, as determined by ELISA. *$p<0.05$. N = 11-18 per group.

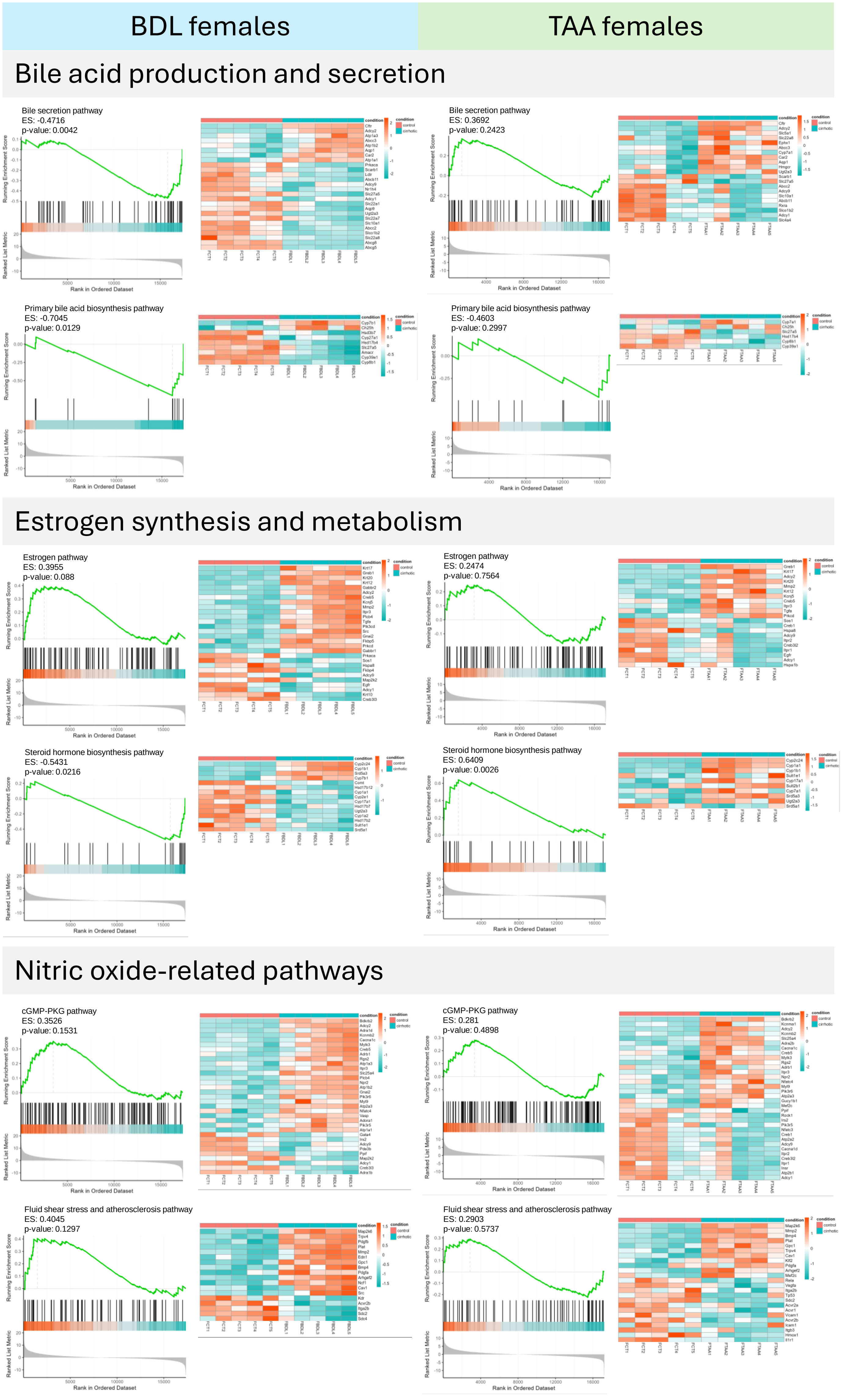

Supplementary figure 4. Gene Set Enrichment Analysis (GSEA) comparing BDL females versus TAA females. The GSEA highlights distinct transcriptomic signatures associated with bile acid production and secretion, estrogen synthesis and metabolism, and nitric oxide-related pathways. Enrichment scores are shown as enrichment scores (ES), with p-value < 0.05 considered significant.

COHORT	NUMBER	SEX	AGE		FIBROSIS STAGE
**MASLD**<br>GSE 162694	**23**	MALE	$\dot{x}$=43.5	<50:14<br>≥50:9	0/1
	**73**	FEMALE	$\dot{x}$=44.7	<50:44<br>>50:29	0/1
	**5**	MALE	$\dot{x}$=59.4	<50:1<br>>50:4	4
	**7**	FEMALE	$\dot{x}$=61.8	<50:0<br>>50:7	4
**HBV**<br>GSE 84044	**48**	MALE	$\dot{x}$=39.4	<50:38<br>>50:10	0/1
	**15**	FEMALE	$\dot{x}$=39.2	<50:13<br>>50:2	0/1
	**8**	MALE	$\dot{x}$=54.6	<50:3<br>≥50:5	4
	**2**	FEMALE	$\dot{x}$=40	<50:2<br>>50:0	4

Supplementary table 1. MASLD and HBV cohorts. The table summarizes the two study cohorts, detailing the number of patients, sex, mean age, and fibrosis stage.

Parameter	Male TAA	Female TAA	p-value	Male BDL	Female BDL	p-value
**AST (U/l)**	135.2 ± 10.62	124.6 ± 9.85	>0.2	1131.8 ± 326.5	1274.7 ± 391.8	>0.2
**ALT (U/l)**	80.8 ± 7.92	76.6 ± 11.48	>0.2	108.8 ± 26.74	65.64 ± 14.40	0.141
**Albumin (g/l)**	29.2 ± 1.33	26.3 ± 0.78	0.068	26.4 ± 2.07	29.5 ± 2.75	>0.2
**GGT (U/l)**	7.5 ± 0.73	10.1 ± 1.22	0.089	158.4 ± 22.91	61.5 ± 7.75	<0.001
**Urea (mg/dL)**	43.1 ± 1.49	43.8 ± 2.42	>0.2	71.5 ± 6.26	83.1 ± 5.48	0.157
**Uric acid (mg/dL)**	2.82 ± 0.28	2.78 ± 0.23	>0.2	2.29 ± 0.25	3.16 ± 0.62	>0.2
**Creatinine (mg/dL)**	1.10 ± 0.05	1.03 ± 0.07	>0.2	1.80 ± 0.38	1.44 ± 0.08	>0.2
**Total bilirubin (mg/dL)**	0.58 ± 0.02	0.57 ± 0.03	>0.2	7.51 ± 0.25	5.25 ± 0.35	<0.0001

Supplementary table 2. Sex differences in liver enzymes. AST, ALT, albumin, GGT, urea, uric acid, creatinine and total bilirubin levels in serum from cirrhotic rats. Results are expressed as mean ± SEM; n = 11-18 per group. p values correspond to Student's t test. AST: aspartate aminotransferase; ALT: alanine aminotransferase; GGT: gamma-glutamyl transpeptidase.

TAA	FUNCTION	♀	♂
Serpina3m	INFLAMMATION	UP	DOWN
Akr1c1	DETOXIFICATION/HORMONAL	UP	DOWN
Hsd11b1	METABOLISM	UP	DOWN
Akr1c12	DETOXIFICATION/HORMONAL	UP	DOWN
Gstt3	METABOLISM	UP	DOWN
Ciart	NEURONAL	UP	DOWN
Per2	NEURONAL	UP	DOWN
RGD1565355 (CD36)	METABOLISM/INFLAMMATION	DOWN	UP
Il17b	INFLAMMATION	DOWN	UP
Vdr	METABOLISM	DOWN	UP
Akr1b7	DETOXIFICATION	DOWN	UP
Prlr	HORMONAL	DOWN	UP
**BDL**	**FUNCTION**	**♀**	**♂**
Akr1c2	DETOXIFICATION/HORMONAL	UP	DOWN
Csad	METABOLISM	UP	DOWN
Hsd11b1	METABOLISM	UP	DOWN
Abcd2	METABOLISM	DOWN	UP
RGD1565355 (CD36)	METABOLISM/INFLAMMATION	DOWN	UP
Fgfr2	PLEIOTROPIC	DOWN	UP
Trim24	METABOLISM	DOWN	UP
Tnfaip6	INFLAMMATION/FIBROSIS	DOWN	UP
Abhd3	METABOLISM	DOWN	UP
Cyp2c12	DETOXIFICATION	DOWN	UP
Mlc1	NEURONAL	DOWN	UP
**HUMAN MASLD**	**FUNCTION**	**♀**	**♂**
SGPP2	METABOLISM	UP	DOWN
GALNT15	METABOLISM	UP	DOWN
SULF1	FIBROSIS	UP	DOWN
HIF3A	METABOLISM	UP	DOWN
MAPK10	NEURONAL	UP	DOWN
LAMA4	FIBROSIS	UP	DOWN
IL17RD	INFLAMMATION	UP	DOWN
SORD	METABOLISM	DOWN	UP
ADH4	METABOLISM	DOWN	UP
ADH1B	METABOLISM	DOWN	UP
AKR1C4	DETOXIFICATION	DOWN	UP
HSD17B6	HORMONAL	DOWN	UP
GSTA1	DETOXIFICATION	DOWN	UP
ADH1A	METABOLISM	DOWN	UP
GSTZ1	DETOXIFICATION	DOWN	UP
ALDH2	METABOLISM	DOWN	UP
MAT1A	METABOLISM	DOWN	UP
LPA	METABOLISM	DOWN	UP
GNMT	METABOLISM	DOWN	UP
UGT2B7	DETOXIFICATION	DOWN	UP
UGT1A4	DETOXIFICATION	DOWN	UP
ALDH6A1	DETOXIFICATION	DOWN	UP
ADH1C	METABOLISM	DOWN	UP
HMGCS2	METABOLISM	DOWN	UP
SLC6A12	NEURONAL	DOWN	UP
ELOVL6	METABOLISM	DOWN	UP
SULT1A2	DETOXIFICATION	DOWN	UP
LCAT	METABOLISM	DOWN	UP
UGT2B17	DETOXIFICATION	DOWN	UP
SLC22A1	DETOXIFICATION	DOWN	UP
CFHR4	INFLAMMATION	DOWN	UP
CFHR5	INFLAMMATION	DOWN	UP
DCXR	DETOXIFICATION	DOWN	UP

Supplementary table 3. Identification of candidates of sexually dimorphic genes for advanced chronic liver disease. The table summarizes potential candidate genes with sexual dimorphism that are suggested to play a significant role in ACLD, their functions, and the direction of gene regulation in each sex. ♀: Female; ♂: male.
